# Observing end-state comfort favorable actions does not modulate action plan recall

**DOI:** 10.3389/fpsyg.2015.00045

**Published:** 2015-01-29

**Authors:** Christian Seegelke

**Affiliations:** ^1^Neurocognition and Action Research Group – Faculty of Psychology and Sport Science, Bielefeld UniversityBielefeld, Germany; ^2^Center of Excellence Cognitive Interaction TechnologyBielefeld, Germany

**Keywords:** action observation, grasping, motor planning, end-state comfort, imitation, representation, action simulation

## Abstract

A large corpus of work demonstrates that observing other people’s actions activates corresponding motor representations in the observer by running an internal simulation of the observed action. Recent evidence suggests that recalled action plans reflect a plan of how the observer would execute that action (based on the specific motor representation) rather than a plan of the actually observed action (based on the visual representation). This study examined whether people would recall an action plan based on a visual representation if the observed movement is biomechanically favorable for their own subsequent action. Participants performed an object manipulation task alongside a confederate. In the intra-individual task, the participant (or confederate) transported a plunger from an outer platform of fixed height to a center target platform located at different heights (home-to-target move), and then the same person transported the plunger back to the outer platform (target-back-to-home move). In the inter-individual task, the sequence was split between the two persons such that the participant (or confederate) performed the home-to-target move and the other person performed the target-back-to-home move. Importantly, the confederate always grasped the plunger at the same height. This grasp height was designated such that if participants would copy the action (i.e., grasp the object at the same height) it would place the participant’s arm in a comfortable position at the end of the target-back-to-home move (i.e., end-state comfort). Results show that participants’ grasp height was inversely related to center target height and similar regardless of direction (home-to-target vs. target-back-to-home move) and task (intra- vs. inter-individual). In addition, during the inter-individual task, participant’s target-back-to-home grasp height was correlated with their own, but not with the confederate’s grasp height during the home-to-target moves. These findings provide evidence that observing actions that are biomechanically favorable for subsequent action execution does not influence action plan recall processes.

## INTRODUCTION

There is convincing evidence to suggest that action planning is contingent upon upcoming task demands or the intended action goals (e.g., Rosenbaum et al., 1990; Cohen and Rosenbaum, 2004; Ansuini et al., 2008; Sartori et al., 2011; Seegelke et al., 2011, 2013a). In one study (Cohen and Rosenbaum, 2004), participants grasped a plunger from a home platform and placed it at one of five target platforms (home-to-target moves). The height of the home platform was fixed whereas the target platforms were located at different heights (two target platforms were lower than the home platform, one platform was at the same height as the home platform, and two platforms were higher than the home platform). It was found that participants changed the height at which they grasped the plunger from the home platform depending on the height of the target platform such that the higher the plunger was to be placed the lower it was initially grasped (and vice versa). Complementing previous findings (cf. Rosenbaum et al., 2012), this inverse relationship between target height and initial grasp height was taken as evidence that participants planned their actions based on future task demands (i.e., target platform height) and adopted initial grasps that would afford comfortable or biomechanically favorable postures at the end, rather than at the start, of the movement (i.e., end-state comfort).

The study of Cohen and Rosenbaum (2004) also demonstrated that action planning is not only influenced by upcoming task demands, but also by previously performed actions (see also Rosenbaum and Jorgensen, 1992; Jax and Rosenbaum, 2007; van der Wel et al., 2007; Weigelt et al., 2009; Schütz et al., 2011; Schütz and Schack, 2013). Specifically, after participants had placed the plunger at the target platform, they lowered their arm, and then transported the plunger back to the home platform (target-back-to-home moves). Interestingly, for these target-back-to-home moves, the height that participants grasped the plunger was very similar to the grasp height of the home-to-target moves, and hence, varied as a function of target platform height. Cohen and Rosenbaum (2004) reasoned that if participants would have planned their target-back-to-home moves *solely* based on future task demands (i.e., home platform height) so as to ensure end-state comfort, this should have resulted in similar grasp heights upon completion of the target-back-to-home moves regardless of target platform height at which the plunger was located (as the home platform was located at a fixed height), thus yielding a zero slope. However, given the similarity of grasp height between the home-to-target and target-back-to-home moves, the authors postulated that participants generated an action plan for the home-to-target moves, and then recalled and slightly modified that plan for the target-back-to-home moves. The authors argued that relying on memory based recall processes reduced the cognitive burden associated with generating a new action plan from scratch.

Recently, Seegelke et al. (2013b) extended the grasping and placing task of Cohen and Rosenbaum (2004) to a social interaction scenario to examine whether the observation of an action is sufficient to elicit plan recall processes. In this study, either a single participant performed the home-to-target and the target-back-to-home moves (intra-individual task) or one participant performed the home-to-target move (while another participant observed the action) and the other participant carried out the target-back-to-home move (inter-individual task). In addition to replicating the results of Cohen and Rosenbaum (2004), this study demonstrated that grasp height during the target-back-to-home move was similar regardless of whether participants had previously performed the home-to-target move (intra-individual task) or whether they had only observed the other participant performing that action (inter-individual task). Complementing the large body of research demonstrating that the observation of an action activates corresponding motor representations in the observer by internally simulating the action (Gallese and Goldman, 1998; Jeannerod, 2001), these findings demonstrate that a simulated action plan can be held in memory and reused for an actor’s subsequent actions. In addition, the results of Seegelke et al. (2013b) provided insights into the nature of a recalled action plan. Specifically, the authors found that participant’s target-back-to-home move grasp height was similar to their own, but not to their partner’s home-to-target move grasp height. Thus, for the target-back-to-home moves participants did not copy the exact grasp height from their partner, but instead grasped the plunger at the height at which they would have grasped it during the home-to-target moves, indicating that a recalled action plan is based on the specific motor representation rather than on the visual representation of the observed action.

As in this study there were considerable differences in grasp height adjustment between participants, the authors argued that if participants in their study were prone to imitate (i.e., to exactly copy) the kinematic parameters of the observed action (i.e., relying on a visual representation), they might have had to adopt quite uncomfortable postures. In contrast, an action plan which is based on the observer’s motor representations takes into account the observer’s physical competencies. Thus, recalling such an action plan ensures successful task completion without putting the limbs into positions that are too awkward or uncomfortable. Consequently, recall of an action plan based on one’s own specific motor representation might be cognitively and biomechanically advantageous. Based on this inference, the present study addressed the question as to whether participants would exhibit a stronger tendency to copy an observed action (i.e., action plan recall based on a visual representation) if the observed movement is biomechanically favorable for one’s own subsequent action (i.e., if copying the observed action leads to end-state comfort satisfaction).

To test this hypothesis, the same experimental task as in Seegelke et al. (2013b) was used except that participants performed the actions alongside a confederate. Importantly, the confederate was instructed to always grasp the object at the same height (i.e., yielding zero slopes). Crucially, this grasp height was designated such that if participants would copy the action (i.e., adopt the same grasp height) during the inter-individual task, it would place the participant’s arm in a comfortable position at the end of the target-back-to-home moves (i.e., end-state comfort). Consequently, if action plan recall processes are modulated by observing actions that are biomechanically favorable for subsequent action execution it was expected that, during the inter-individual task, participants’ target-back-to-home move grasp height should be similar to the confederate’s home-to-target move grasp height, thus yielding zero, or close to zero, slopes.

## MATERIALS AND METHODS

### PARTICIPANTS

Twenty-four individuals (mean age = 25.09 years, SD = 2.95, 14 female, 10 male) participated in exchange for 5€ or course credit. All participants were right handed as assessed using the Revised Edinburgh Inventory (Dragovich, 2004; mean score = 98.63, SD = 6.74), had normal or corrected to normal vision, and were physically and neurologically healthy. The experiment was conducted in accordance with local ethical guidelines, and conformed to the declaration of Helsinki. All participants gave their informed written consent to participate in the study. The confederate was male, right-handed, 27 years old, and 174 cm tall.

### APPARATUS

**FIGURE 1:**
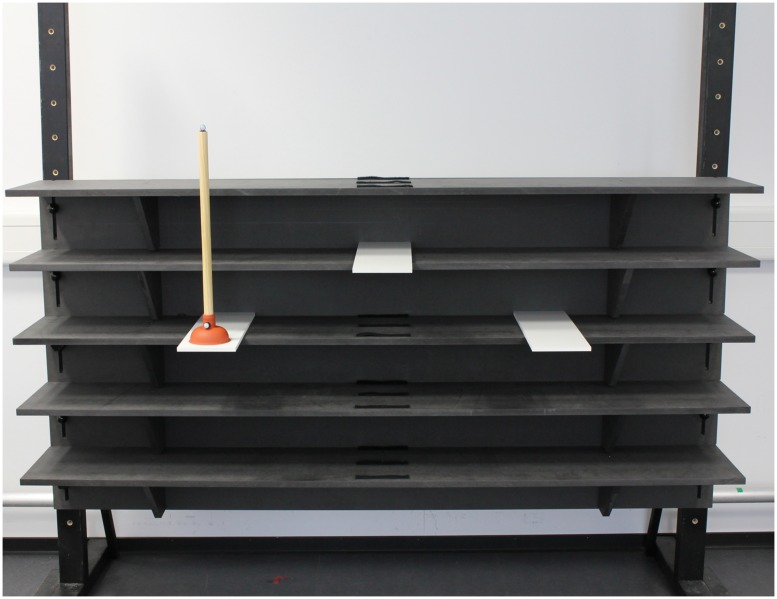
**Experimental setup**.

The experimental apparatus was identical to that used in Seegelke et al. (2013b). It consisted of five wooden shelves (200 × 30 cm) located at 50 cm, 70 cm, 90 cm, 110 cm, and 130 cm height, fixated by two legs (**Figure 1**). Two outer platforms (45 × 15 cm) were attached to the 90 cm shelf, positioned 45 cm to either side of the shelf’s midpoint, and extended 15 cm from the shelf. The center platform (45 × 15 cm) could be attached centrally to each of the five shelves and extended 15 cm like the outer platforms. The manipulated object was a plunger with a wooden cylindrical shaft (50 cm in height, 2.5 cm in diameter) and a circular rubber base (5 cm in height, 10 cm in diameter).

An optical motion capture system consisting of 10 Bonita cameras (VICON Motion Systems, Oxford, UK) was used to collect kinematic data from retro reflective markers at 200 Hz. Markers were placed on the styloid process of the radius (WRT) on the right hand of the participant and the confederate, and on the base of the plunger shaft (PB).

### PROCEDURE AND DESIGN

After filling out the informed consent and handedness inventory, retro reflective markers were placed on the right hand of the participant and confederate. The participant and confederate were arbitrarily designated A and B, respectively. Participant and confederate stood side by side in front of the outer platforms at a distance of 30 cm from the front edge of the shelves. At the start of each trial, the participant and confederate closed their eyes and placed the hands by their sides. The experimenter then attached the center platform at the respective shelf height, and placed the plunger on one of the outer platforms (left or right), depending on condition. After the experimenter verbally indicated which of the two persons (i.e., A = participant, B = confederate) would perform the first move both persons opened their eyes, and the named one grasped and transported the object with the right hand from the outer platform to the center platform (home-to-target move, outer-to-center) and then placed the hand back to the side of the body. The other person observed the performed action. The experimenter then verbally indicated who would perform the target-back-to-home move (center-to-outer) and the respective person then grasped and transported the object back to the outer platform. The participant and confederate operated only in their workspace. Thus, when the plunger was initially located on the left outer platform the person standing in front of the left outer platform performed the home-to-target move. Similarly, if the person standing in front of the right outer platform performed the target-back-to-home move he or she placed the object on the right outer platform. Thus, in the intra-individual task, either the participant or the confederate performed the home-to-target and the target-back-to-home move while the other watched the action as it was performed. In the inter-individual task, either the participant or the confederate performed the home-to-target move while the other observed, and then the other person performed the target-back-to-home move.

In order to assess participants’ grasp height that would lead to comfortable postures at the end of the target-back-to-home move (i.e., end-state comfort), participants performed six ‘posture comfort assessment trials’ in which they performed both the home-to-target and target-back-to-home move (as in the intra-individual task) with the center platform attached to the 90 cm shelf (three for each outer platform side). This condition was chosen because the same grasp height ensures end-state comfort for both the home-to-target and target-back-to-home moves [i.e., the heights of the home and target platform coincide (i.e., both at 90 cm)]. As such, any influence on grasp height of the target-back-to-home moves from the previous home-to-target move (due to recall processes) can be controlled. After completion of these trials, mean grasp height of the target-back-to-home moves was calculated (see data processing section for details) and the experimenter marked the grasp height by drawing a thin line with a pencil on the object’s shaft at the respective position. For the following experimental trials, the experimenter carefully paid attention that the plunger was placed on the outer platforms such that only the confederate but not the participant could see the marking. Post-experimental questionnaire confirmed that this succeeded as none of the participants noticed the marking on the object.

Participants were instructed to perform the movements at a comfortable speed, and to grasp the plunger firmly such that it would not slip through their fingers during the transport. The confederate was instructed to always grasp the plunger at the marked height irrespective of condition. There were a total of 40 trials per participant which consisted of each possible combination of the factors task (intra-individual and inter-individual), center platform height (50, 70, 90, 110, 130 cm), and object position (left, right). The factor task was blocked and the order of blocks was counterbalanced across participants. Within each task block, participants standing position (i.e., in front of the left or right outer platform) was balanced, and each participant performed two trials for each center platform height in a randomized order. The entire experiment took about 40 min.

### DATA PROCESSING AND ANALYSIS

The 3D coordinates of the retro reflective markers were reconstructed and missing data were interpolated using a cubic spline. The marker coordinates were filtered using a Woltring (1986) filter with a predicted mean square error of 5 mm^2^ (Vicon Nexus). Kinematic variables were calculated using custom written MatLab programs (The MathWorks, Version R2010a). For each trial, the home-to-target move (outer-to-center target) was defined as the time period between when the plunger was lifted from the outer platform to the time the plunger was placed on the center platform. The target-back-to-home move (center-to-outer) was defined as the time period between when the plunger was lifted from the center platform to the time the plunger was placed on the outer platform. Onset of each move was determined as time of the sample in which the resultant velocity of the plunger marker (PB) exceeded 5% of peak velocity of the corresponding move. Offset of each move was determined as the time of the sample in which the resultant velocity dropped and stayed below 5% of peak velocity of the corresponding move. Grasp height was calculated as the vertical distance between WRT and PB (in mm), and extracted when the plunger was located on the outer platform. Thus, grasp height was extracted at the onset of the home-to-target moves and at the offset of the target-back-to-home moves.

Participants’ grasp height data were analyzed using a 2 task (intra-individual, inter-individual) × 2 direction (home-to-target move, target-back-to-home move) × 2 object position (left, right) × 5 center platform height (50 cm, 70 cm, 90 cm, 110 cm, 130 cm) repeated measures analysis of variance (RM ANOVA)^1^. In addition, linear regressions were conducted for the grasp heights on the center shelf heights separately for each task, direction, and object position, and for each participant and each confederate performance. The slopes of the best fitting straight lines provide a good and robust estimate of the degree of grasp posture adjustment to center platform height.

## RESULTS

### CONFEDERATE PERFORMANCE

Confederate’s slopes of the home-to-target move during the inter-individual task were 0.00 for both the left and right object position and did not significantly differ from zero (*p* = 0.915 and *p* = 0.888 for object position left and right, respectively; see **Table 1**), indicating that the confederate did not adjust his grasp height to center platform height. Furthermore, confederate’s grasp height in this condition (i.e., inter-individual home-to-target move) resembled participant’s grasp height during the target-back-to-home moves in the posture comfort assessment task as evidence by the small absolute mean difference (17 mm, SD = 13) between confederate’s home-to-target move inter-individual grasp height and participants’ target-back-to-home move posture comfort assessment grasp height. Thus, these data demonstrate that the confederate followed the instructions to grasp the plunger at the marked height.

**Table 1:** Slopes, intercepts, and correlations (r) for best-fitting straight lines relating grasp height (mm) to center shelf height (mm) in home-to-target and target-back-to-home moves for each task condition.

	Home-to-target move						Target-back-to-home move					
	Object position						Object position					
	Left			Right			Left			Right		
	Slope	Intercept	*r*	Slope	Intercept	*r*	Slope	Intercept	*r*	Slope	Intercept	*r*
**Participants**
Intra-individual	-0.12***	359	0.527	-0.15***	382	0.595	-0.10***	332	0.434	-0.13***	371	0.532
Inter-individual	-0.12***	354	0.473	-0.12***	360	0.489	-0.10**	336	0.403	-0.10***	343	0.399
**Confederate**
Intra-individual	0.00	251	0.004	0.00	256	0.027	0.00	241	0.022	0.00	235	0.026
Inter-individual	0.00	253	0.010	0.00	253	0.013	0.00	239	0.009	0.00	243	0.008

*p < 0.05, **p < 0.01, ***p < 0.001.

### PARTICIPANT PERFORMANCE

**FIGURE 2:**
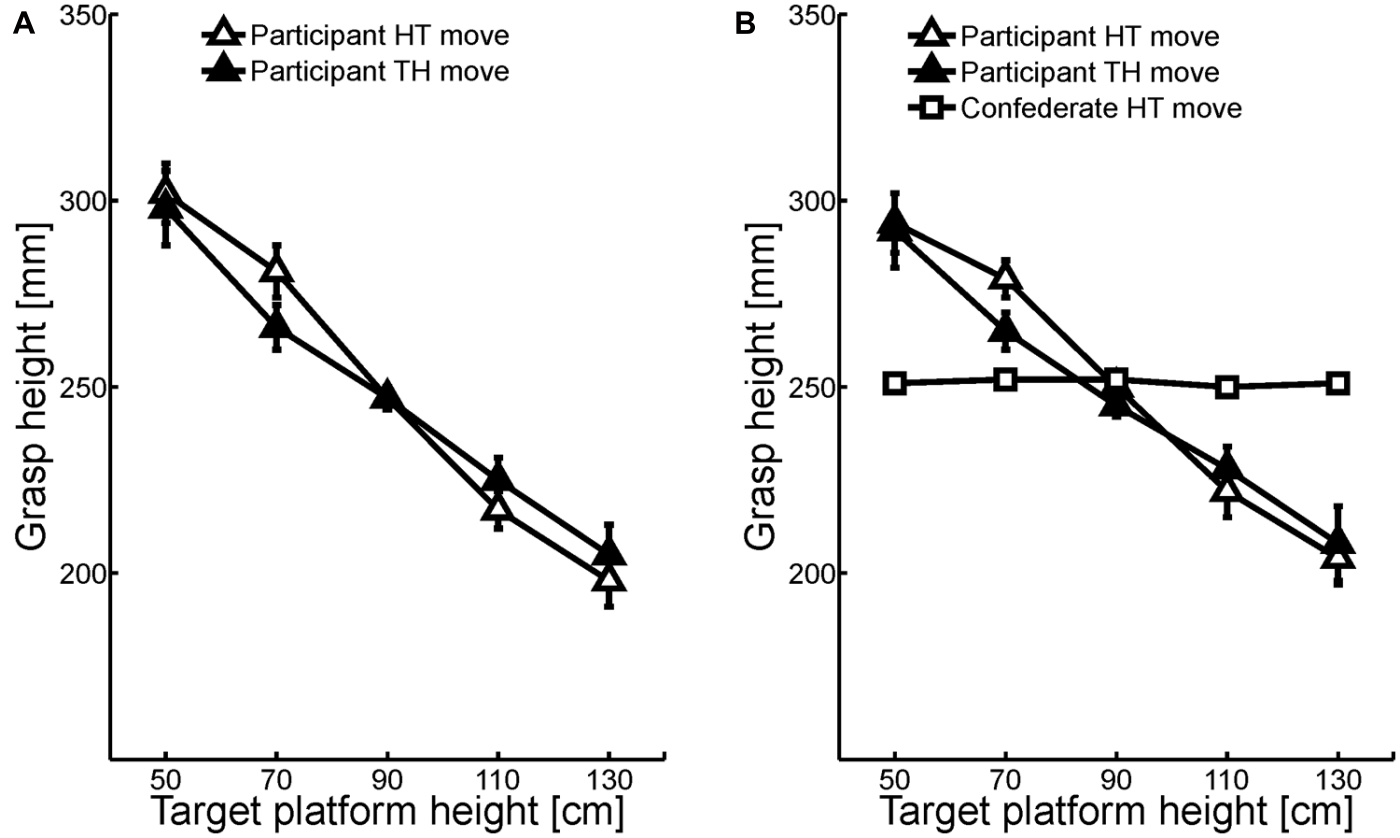
**Mean grasp heights (relative to plunger base) as a function of center target height during the intra-individual task **(A)** and the inter-individual task **(B)** for the home-to-target moves (HT; white marks) and target-back-to-home moves (TH; black marks).** Error bars represent standard errors after removal of between-subject variability (Cosineau, 2005).

The RM ANOVA on participants’ grasp height revealed a significant main effect of object position, *F*(1,23) = 6.08, *p* = 0.022. Participants grasped the plunger higher when it was transported to or from the right outer platform (251 mm) than when it was transported from or to the left outer platform (246 mm). In addition, grasp height was inversely related to center platform height (**Figure 2**), *F*(4,92) = 35.79, *p* < 0.001. Importantly, there was no main effect of task or direction, nor a significant interaction with one of these factors, demonstrating that grasp height was similar for the home-to-target and target-back-to-home moves and for the intra-individual and inter-individual task (**Figure 2**). The slopes of the best-fitting straight lines ranged from -0.10 to -0.15, and all differed significantly from zero (all *p* < 0.001, see **Table 1**).

To further test whether participants’ grasp height was influenced by the confederate’s grasp height, correlation analyses were conducted on the participants’ and the confederate’s slopes during the inter-individual task. Analyses showed that participants’ slopes during the target-back-to-home moves were strongly correlated with participants’ slopes during the home-to-target moves (*r* = 0.80, *p* < 0.001) but not with the slopes of the confederate’s home-to-target moves (*r* = 0.12, *p* = 0.576).

## DISCUSSION

The present study examined whether action plan recall processes are modulated by observing actions that are biomechanically favorable for one’s own subsequent action execution. Specifically, it was expected that participants would be more prone to copy an observed action (i.e., utilizing the visual representation rather than the own specific motor representation) if the recall of the action plan based on this representation would result in comfortable final postures (i.e., end-state comfort). The results do not support this hypothesis. Specifically, during the inter-individual task, participants’ target-back-to-home move grasp height was considerable different from the confederate’s home-to-target move grasp height, and also differed significantly from zero. In addition, a high correlation between participants’ inter-individual target-back-to-home move and participants’ home-to-target move slopes was observed, whereas there was no correlation between participants’ target-back-to-home move and the confederate’s home-to-target move slopes, indicating that action plan recall is based on the observer’s specific motor representation.

A large amount of research has demonstrated that observing other persons’ movements activates the corresponding motor representations in the observer by running an internal simulation of the observed action (Jeannerod, 2001; Flanagan and Johansson, 2003; Grafton, 2009). Extending these findings, a recent study (Seegelke et al., 2013b) indicated that a simulated action plan can be held in memory and recalled for subsequent own actions, and that a recalled action plan resembles a plan of how the observer would execute that action (i.e., based on a motor representation) rather than a plan of the actually observed action (i.e., based on a visual representation). The results of the present study provide complementary evidence for a motor representation-based recall process, regardless of whether a visual representation-based plan recall would have been biomechanically favorable for subsequent action execution.

These findings are somewhat at odds with studies which have demonstrated that (involuntary) copying of actions might occur even if it is detrimental to task performance (Cook et al., 2012; Belot et al., 2013). Importantly, in these studies, participants performed the actions simultaneously, whereas the present task was sequential. Consequently, the time delay between the observed and executed action might have allowed participants to inhibit an imitative tendency. This explanation is indeed supported by recent TMS studies (Sartori et al., 2012, 2013; Barchiesi and Cattaneo, 2013; Cross and Iacoboni, 2014) forwarding the view of a biphasic time course of brain activation to action observation, whereby direct matching mechanisms are engaged in the early phase, and later phase processes consider contextual or intentional information. Although the present study does not provide a direct measure of internal action simulation processes during action observation, it is certainly possible that participants’ internal action simulation relied on a visual representation, but that this representation was further inhibited or suppressed, such that the recalled action plan was based on the participants’ specific motor representation.

An alternative, though not mutual exclusive, interpretation that participants did not imitate (i.e., exactly copy) the kinematic parameters of the confederate’s action despite the fact that it would have ensured a comfortable final posture might be that participants do not solely plan their movements to satisfy end-state comfort. For example, in Seegelke et al. (2013b), there was also a condition in which a single participant performed the entire action sequence but in a reversed order such that they first transported the plunger from a platform of varying height to a platform of fixed height, and vice versa for the return move. Although in this condition, slopes of the first (variable-to-fixed) moves were considerably shallower compared to the other conditions, they still deviated significantly from zero, indicating that participants assigned some degree of comfort to the start of the movement. Indeed, more recent research has demonstrated that additional factors are taken into account during the planning of sequential object manipulation tasks (e.g., Seegelke et al., 2011, 2012, 2013a,b; Hughes et al., 2012; Herbort et al., 2014; Seegelke et al., 2015) suggesting that motor planning during sequential actions is guided by a constraint hierarchy in which specific constraints are weighted relative to each other for successful task performance. In this context, the task might have been also too easy or less demanding (with respect to biomechanical constraints) to elicit that participants would copy the observed action. To increase task difficulty, Rosenbaum et al. (2006) employed the Cohen and Rosenbaum (2004) task and manipulated the precision demands at the start and the end of the movement. In addition to replicating the original results, the authors found that participants grasped the plunger closer to its base when the precision demands of the task were high. Rosenbaum et al. (2006) concluded that grasping the plunger lower when high precision was required was rationale from a biomechanical perspective, as this allows for better controlling the orientation of the plunger. As the precision demands in the present study were comparable low, biomechanical constraints were negligible and hence, participants could accomplish the task goal without the necessity to adopt end-state comfort compliant grasp postures.

This reasoning also fits with the concepts of rationale action and teleological reasoning (e.g., Gergely et al., 2002; Gergely and Csibra, 2003; Csibra and Gergely, 2007). These frameworks propose that infants of ∼12 months of age are capable of interpreting other’s actions as means to goals and evaluate the rationality of the means with respect to the task goal and situational constraints. For example, Gergely et al. (2002) demonstrated that 14-month-old children will only copy an adult demonstrator’s action means to achieve the same goal if they consider it the most rational alternative. If the observed behavior can be rationalized by the model’s situational constraints, but the infant has different constraints, they will achieve the same action goal by the most rational means under consideration of their own situational constraints. As such, for the experimental task of the present study, it is possible that changing the situational constraints of the observer (for example by increasing the final precision demands) will modulate the action means (i.e., grasp height) by which the observer will achieve the task goal (i.e., placing the plunger back at the home platform), and hence, increase the tendency to copy the confederate’s action means.

In sum, the data from the present experiment provide further evidence that, during sequential grasp-to-place actions, recalled action plans are based on the observer’s specific representation and do not resemble a direct copy of the observed action, even if action plan recall based on a visual representation would have been biomechanically favorable with regards to the observer’s subsequent action execution. Future research should examine whether changing the observer’s situational (i.e., biomechanical) constraints will modulate the observer’s action choices.

## Conflict of Interest Statement

The author declares that the research was conducted in the absence of any commercial or financial relationships that could be construed as a potential conflict of interest.

## Footnotes

1 Given that gender can affect cooperation (see Balliet et al., 2011, for a review) and the confederate was always male, data were also analyzed including only female or only male participants. Both analyses yielded the same pattern of results indicating that gender did not affect performance in the present study.
